# Tumors

**Published:** 1828-08

**Authors:** 


### TUMORS.

### *Cases of Tumors removed from different Parts of the Body.*
By Dr. Ballingall.

(Concluded from page 56.)

V.—James M'Intosh was admitted, November 12th, with a soft moveable tumor impacted between the roof of the orbit and globe of the right eye; the superior eyelid was protruded outwards and considerably inflamed, as well as the conjunctiva covering the surface of the tumor; the ball of the eye was depressed by the swelling towards the cheek. The structure of the eye, however, appeared perfectly sound, and the vision unimpaired, except in so far as it was partially obstructed by the projection of the tumor, which obliged the patient to throw back his head, and to elevate his face in attempting to see objects placed before him. He was unconscious of any accident to which this complaint could be attributed, assigning its origin to exposure to cold in the month of January preceding. This patient was in the hospital in the month of July last, at which time the tumor was not above a fourth part of its present size, and occupied nearly the site of the lachrymal gland: he was urged to have it removed, but would not consent, although told that he would in all probability return with it at a future period, when the operation would be more difficult both for him and for me.

This accordingly happened, and he was now solicitous for its removal, which Dr. Ballinghall began by dividing the superior palpebræ upwards and outwards from the external canthus of the eye, and, after dissecting it off from the surface of the swelling, the tumor was with much difficulty separated from the contiguous parts; a pedicle, or neck, by which it was found adherent to the very bottom of the orbit, was then cut across with a pair of probe-pointed scissors, and some small portions of it afterwards removed.

This was followed, in the first instance, by a very moderate degree of swelling and inflammation; much less, indeed, than was to be anticipated. For nearly a week the case had a very favorable aspect, but at the end of this time the forehead and upper part of the face became involved in a violent erysipelatous inflammation, which gradually extended over the whole head, accompanied with delirium, his pulse at one time rising as high as 150. It was observed, soon after the operation, that his breath was imbued with the mercurial fetor, which he attributed to some medicines taken before his admission. The urgent symptoms were somewhat alleviated by bleeding, both general and topical, by the internal exhibition of antimonials and saline purgatives, the application of a blister to the nape of the neck, with the use of an anodyne fomentation to the inflamed parts.

On the 22d, he was found to have sunk so low that he was not expected to live through the ensuing night; his pulse 120, his breathing laborious, and his extremities cold, with low muttering typhoid delirium. From this state he again rallied under the use of brandy-and-water, beef-tea, and the application of a second blister to the nape of the neck. A copious discharge of unhealthy matter had for some days been going on from the affected eye, the cornea of which now ulcerated; and, on the morning of the 27th, the crystalline lens was discharged through the aperture. His delirium continued with occasional intermissions, during which he asked for and devoured food with a ravenous appetite. His pulse continued frequent and weak, his breath fetid and offensive, and his general appearance resembling that of a patient in the advanced stages of typhus. The cuticle separated in crusts from those parts of the head and face in which the inflammation had been seated; rigors and diarrhœa latterly supervened; and he expired on the evening of the 28th.

Permission could not be obtained to examine the body, and the utmost the surgeons could effect was to make a hasty examination of the head and the parts concerned in the operation. A portion of the principal tumor was found still adherent to the sheath of the optic nerve, and several small melanotic tubercles imbedded in the fatty matter surrounding the muscles of the eye. Some serous effusion had taken place both on the surface and into the ventricles of the brain.

Dr. Ballingall states, that if he had been fully aware of the nature of the disease, and of the deep attachment of the tumor, he should have proceeded at once to extirpate the whole contents of the orbit; but having succeeded in removing the bulk of the tumor with safety to the eyeball, he felt reluctant to change his plan of operation. The inflammation immediately succeeding to the removal of the tumor was much less than was to have been expected from so severe an operation; but, when the symptoms of erysipelas supervened, it was obvious that the case became one of a very perplexing and hazardous description.

VI.—Robert Amos was admitted on the 29th of January. A soft elastic tumor, slightly moveable, about the size of an orange, projects from the right orbit; superiorly, the finger may be readily insinuated between it and the skin of the eyebrow; but inferiorly, internally and externally, it encroaches much on the skin of the surrounding parts; the skin of the palpebræ and the conjunctiva, which cover the greatest part of its surface, are (particularly the former) of a dark colour, and the veins of these membranes are much enlarged and very tortuous; in the centre of the tumor there is a sloughy bleeding fungus, slightly elevated above the remainder of the swelling, and through this the probe may be passed backwards for about two inches and a half: the passage of the probe is attended by some thick dark-coloured fetid discharge. On the right and left temple, and under the skin of the under and upper lip, there are smaller tumors of a similar character, but much firmer in consistence. Ten years ago the eye was injured by a blow, two years afterwards the sight was completely lost, and in six years more lancinating pain began, and was succeeded by the swelling, which has continued to increase for the last twenty-two months.

The whole of this diseased mass was removed a few days after his admission, and the bony margin of the orbit, near the external canthus of the eye, being found affected by the disease, a portion of it was scraped off with a strong scalpel. The cavity was afterwards filled with dry lint, which readily suppressed the hemorrhage.

This man's symptoms, subsequent to the operation, were extremely mild: the cavity suppurated kindly, and its surface soon became covered with healthy, perhaps rather pale-coloured, granulations. About ten days after the operation, he had (like the other cases of operation within the orbit) a smart attack of erysipelas, which disappeared in a few days under the employment of bloodletting and purgatives. The last report states, that the man's general health continues apparently unimpaired, and the interior surface of the orbit presents a smooth, healthy looking surface, the discharge from which is daily diminishing.

On examining the eye, the texture of the tumor was found so completely broken down as to render it difficult to speak with certainty as to its original nature. Dr. Ballingall, however, is disposed to consider it as an example of the fungus hæmatodes. This tumor began, as far as could be ascertained, in the globe of the eye: it presented the soft elastic feel characteristic of fungus hæmatodes; it was attended with lancinating pains, and was subject to occasional bleedings. The principal, and perhaps the only argument against this opinion as to its nature, is the existence of other tumors in the neighbourhood, evidently of a melanotic appearance; a circumstance which goes to support the identity of the two affections.*

* Condensed from Dr. Ballingall's Clinical Lectures, March 1828.

